# The Incidence and Prevalence of Eating Disorders Between 1975 and 2024: A Commentary on Lee and Chi (2025)

**DOI:** 10.1002/eat.24495

**Published:** 2025-07-02

**Authors:** Hans W. Hoek

**Affiliations:** ^1^ University of Groningen University Medical Center Groningen Groningen the Netherlands; ^2^ Columbia University Mailman School of Public Health New York New York USA; ^3^ Parnassia Psychiatric Institute The Hague the Netherlands

**Keywords:** anorexia nervosa, ARFID, bulimia nervosa, eating disorders, incidence, males, older persons, prevalence, social media, transcultural

## Abstract

Over the past five decades (1975–2024), research on eating disorders (EDs) has expanded significantly, as evidenced by the bibliometric analysis by Lee and Chi (2025). This growth reflects heightened public and academic interest, likely influenced by several key developments in the classification and epidemiology of EDs. The Diagnostic and Statistical Manual for Mental Disorders has progressively included more ED diagnoses, such as bulimia nervosa, binge‐eating disorder (BED), and avoidant/restrictive food intake disorder (ARFID) between 1975 and 2024. Recognition of EDs among males and older individuals has improved, although these groups remain underrepresented in clinical settings and in research. Global studies indicate rising ED prevalence in Asian countries. While anorexia nervosa remains relatively rare in Latin America and Africa, bulimia nervosa and BED are also common disorders on these continents. Epidemiological data in the Netherlands suggest that, overall, ED incidence has not increased between 1975 and 2024; however, a notable exception is the significant increase in anorexia nervosa among 10‐ to 14‐year‐old girls. Emerging evidence indicates that since the onset of and during the COVID‐19 pandemic, there has been a global rise in reported cases of EDs.

## 1 Introduction

In their bibliometric analysis Lee and Chi (2025) examined publication trends on eating disorders (EDs) between 1975 and 2024. Over these five decades, the field of EDs has experienced significant growth in both public engagement and academic attention. Their analysis revealed a marked increase in the number of publications, from 161 in 1975 to 7152 in 2024. This surge might reflect a general acceleration in research activity, but could also be at least partly attributed to changes in the classification and epidemiology of EDs during this time. Our research group has studied the epidemiology of EDs over the same five decades as the study by Lee and Chi. In this commentary and referring to some of our key publications on this subject, we will address five key developments in the occurrence of EDs that may have contributed to the rise in publications and public interest:

1. Expansion of EDs classification in the *Diagnostic and Statistical Manual of Mental Disorders (DSM)*
2. EDs among males and older persons
3. Worldwide occurrence of EDs
4. Changes in the incidence of EDs
5. The impact of the COVID‐19 pandemic on EDs

## 2 Expansion of DSM‐Classified Eating Disorders

Anorexia nervosa was already well‐characterized and named in the 19th century and was included in the first edition of the DSM in 1952. Bulimia nervosa did not appear in the scientific literature until 1979. In 1980, it was recognized and included as “bulimia” in DSM‐III, and from DSM‐III‐R in 1987 onwards, it was referred to as bulimia nervosa. DSM‐IV, published in 1994, introduced several important changes to the categorization of EDs. First, EDs were placed in a separate section, which included anorexia nervosa and bulimia nervosa plus a residual category, eating disorder not otherwise specified (EDNOS). Draft criteria for binge‐eating disorder (BED) were included in an appendix providing criteria sets for further study. In 2013, DSM‐5 was published and BED was recognized as a distinct ED. Additionally, avoidant/restrictive food intake disorder (ARFID) was included in the section on EDs and the name of the DSM‐5 section was changed to “Feeding and Eating Disorders.”

In summary, the number of specified EDs has increased over time and new disorders generally generate significant academic attention.

## 3 Eating Disorders Among Males and Older Persons

As Lee and Chi (2025) note, research on male populations exhibited a strong negative association with both Altmetric Attention Scores per Year (AASPY) and citations per year (CPY), indicating that studies on males with EDs received less public and academic attention than those on females. Several factors may contribute to this trend. Historically, EDs in men have been underdiagnosed and underreported, leading to fewer studies and lower overall research volume (Lee and Chi 2025).

DSM‐5 broadened the diagnostic criteria for anorexia nervosa and bulimia nervosa and officially recognized BED as a distinct diagnosis. These changes are particularly relevant for males and older females, whose ED symptoms tend not to fit into stringent categories. Compared to the DSM‐IV, DSM‐5 allows more EDs in males to be identified by a specific diagnosis rather than a residual category such as EDNOS. For instance, the removal of the amenorrhea requirement and more permissive wording of the weight criterion might facilitate a diagnosis of anorexia nervosa in males.

Males with EDs are rare in clinical settings. It is clear from clinical and epidemiological studies that there are males with EDs, but they are usually much harder to detect than females with EDs. This underdetection may result from a double stigma for males: the general stigma associated with mental disorders and an additional stigma, stemming from the perception of EDs as ‘female‐specific’ conditions.

Older persons with EDs are also infrequently seen in specialized care and there remains a paucity of clinical studies focusing on patients in middle or older age. Nevertheless, empirical evidence indicates that EDs and disordered eating do occur in these cohorts (Mangweth‐Matzek et al. 2023). One important factor contributing to the lower rates of older individuals seeking specific ED treatment may be the overlap with aging‐related symptoms, as well as experiences of stigmatization and shame. While initial studies on older women were based on case reports and small samples, more recent research includes larger sample sizes, encompassing individuals aged 40 to 80 years. These studies reveal that, among older persons, the prevalence of EDs based on DSM‐IV or DSM‐5 criteria ranges between 2% and 7% for women and less than 1% for older men; the prevalence of EDs decreases by age in women, but it does not reach zero even in very high age (Mangweth‐Matzek et al. 2023). Menopausal transition in midlife can be as significant for the development of EDs as puberty is during adolescence. Studies have reported higher prevalence of EDs in perimenopausal women compared to pre‐ or postmenopausal women.

## 4 Worldwide Occurrence of Eating Disorders

In DSM‐5, Criterion B for anorexia nervosa was expanded to include not only an intense fear of gaining weight or becoming fat, as in DSM‐IV, but also persistent behavior that interferes with weight gain. This clarification allows for the inclusion of individuals who may not overtly express fear of weight gain, but exhibit behaviors indicative of such fears, such as active food avoidance or restriction. This is particularly relevant in certain Asian countries, including China, Japan, and regions in South Asia, where cultural factors may influence the expression of ED symptoms.

Recent studies indicate that EDs are present in Asia and suggest an increasing prevalence among young females. In China and Japan, EDs including anorexia nervosa might be as common as in Europe (Hoek 2016).

In contrast, anorexia nervosa remains relatively rare in Africa and Latin America. However, bulimia nervosa and especially BED are common mental disorders in both Latin‐America and Africa (Hoek 2016). Cultural factors, such as body ideals that favor a more curvaceous shape and higher body weight, may offer some protection against the development of anorexia nervosa in these populations.

## 5 Changes in the Incidence of Eating Disorders

Most epidemiological data on EDs worldwide are based on prevalence studies. However, to study time trends, incidence rates—which measure the number of new cases in a population per year—are more informative. Due to the relatively low incidence of EDs in the general population, such studies are challenging, and few incidence studies exist. Consequently, incidence rates have often been derived from hospital records and case‐registers of inpatients and outpatients in mental healthcare facilities. Time trend data suggest an increase in the incidence of anorexia nervosa in mental healthcare settings in Northwestern Europe from the 1930s into the 1970s (Hoek 2016). This rise may reflect improved detection and the availability of more mental healthcare facilities, but it could also indicate a true increase in incidence during that period. One of the suggested explanations for this rise till the 1970s was the adoption of another beauty ideal in the 1960s and 1970s, as represented by very thin models such as the famous supermodel Twiggy in the mid‐sixties. From 1970 into the 21st century, the incidence of anorexia nervosa in mental healthcare facilities in the Netherlands appeared to stabilize at around 5 per 100,000 total population per year (Hoek 2016; Van Eeden et al. 2023).

To study long‐term patterns in the development of EDs at a level that comes closer to the true incidence in the population than the rates in mental health care, the incidence in primary care—the first level of formal detection within the healthcare system—was studied in The Netherlands. A nationwide network of general practitioners, serving approximately 1% of the total Dutch population, recorded newly diagnosed patients with anorexia nervosa and bulimia nervosa in their practices from 1985 till 2024. Time trends of BED and ARFID could not be studied in this primary care study, because they have only been officially classified as specific feeding and EDs in DSM‐5 in 2013.

In the 1980s, 1990s, 2000s, and 2010s, the incidence of anorexia nervosa in primary care was stable, ranging between 6 and 8 per 100,000 total population per year (Van Eeden et al. 2023). In contrast with anorexia nervosa, the incidence of bulimia nervosa has declined significantly over these four decades, from nearly 9 in the 1980s to 3 per 100,000 total population per year in the 2000s and 2010s (Van Eeden et al. 2023). While the overall incidence of anorexia nervosa in the population was rather stable, the incidence of anorexia nervosa among 10‐ to 14‐year‐old girls did increase significantly, from 9 to 39 per 100,000 person‐years over these four decades, suggesting a shift toward earlier onset. Both biological and sociocultural factors, such as earlier pubertal timing and the high impact of social media on young adolescents, particularly in the 2010s, may explain this trend (Van Eeden et al. 2023).

Lee and Chi (2025) also note that a key factor contributing to higher public engagement on EDs among adolescents and young adults is their predominant use of digital platforms, including social media, news websites, and online discussion forums. Adolescents are among the most active users of online media, where topics related to EDs, body image, diet culture, and mental health frequently trend.

## 6 The Impact of the COVID‐19 Pandemic

The COVID‐19 pandemic has had a profound impact on societies worldwide in the early 2020s. It introduced unprecedented stressors, particularly for individuals at risk for or already experiencing EDs. These stressors included widespread uncertainty and social isolation, reduced access to social support networks, disruptions to daily routines (including limitations on physical activity), and restricted access to mental health services (Meier et al. 2025). These factors are known to exacerbate the risk factors for developing an ED. Heightened stress and anxiety during the pandemic may have resulted in a greater perceived loss of control and triggered maladaptive coping mechanisms, such as disordered eating behaviors. Increased social media usage and therefore increased exposure to idealized body images on social media may intensify body dissatisfaction, a key ED risk factor. Emerging evidence indicates that since the onset of and during the COVID‐19 pandemic, there has been a global rise in reported cases of EDs, particularly anorexia nervosa and bulimia nervosa, along with increased healthcare utilization for these conditions (Meier et al. 2025).

In our Dutch primary care study, we could compare the incidence of EDs in the in‐pandemic period (2020–2022) with the pre‐pandemic period (2015–2019). The overall incidence of anorexia nervosa increased (by 29%), but not statistically significant across the two periods in the Netherlands (Meier et al. 2025). Although the incidence of anorexia nervosa among girls aged 10–14 further increased during the COVID‐19 pandemic, this increase was also not statistically significant.

## 7 Conclusions

Between 1975 and 2024, clinicians and researchers in the field of EDs have increasingly recognized that these conditions extend beyond anorexia nervosa alone. Numerous epidemiological findings have demonstrated increased awareness and improved detection of EDs globally, over time, and among diverse populations beyond the traditionally high‐risk category of young females in Western countries.

The incidence of anorexia nervosa in mental healthcare settings has increased in the past century until the 1970s. However, there is little evidence to suggest a significant increase in the overall incidence of EDs in the period 1975–2024, with the notable exception of a significant rise in anorexia nervosa cases among girls aged 10 to 14 years.

Substantial developments in the field over the past five decades, as outlined in the bibliometric analysis by Lee and Chi (2025), encompass not only trends in scientific publication and public interest but also advancements in the classification and epidemiology of EDs. The acknowledgement that EDs not only affect young females, but also males and older individuals, and exist in non‐Western societies has deepened our understanding of these complex mental disorders.

## Author Contributions

**Hans W. Hoek:** conceptualization, writing – original draft, writing – review and editing.

## Conflicts of Interest

The author declares no conflicts of interest.

## Data Availability

Data sharing is not applicable to this article as no new data were created or analyzed in this study.
